# Three-Dimensional Spectral-Domain Optical Coherence Tomography Data Analysis for Glaucoma Detection

**DOI:** 10.1371/journal.pone.0055476

**Published:** 2013-02-11

**Authors:** Juan Xu, Hiroshi Ishikawa, Gadi Wollstein, Richard A. Bilonick, Lindsey S. Folio, Zach Nadler, Larry Kagemann, Joel S. Schuman

**Affiliations:** 1 University of Pittsburgh Medical Center (UPMC) Eye Center, Eye and Ear Institute, Ophthalmology and Visual Science Research Center, Department of Ophthalmology, University of Pittsburgh School of Medicine, Pittsburgh, Pennsylvania, United States of America; 2 Department of Bioengineering, Swanson School of Engineering, University of Pittsburgh, Pittsburgh, Pennsylvania, United States of America; Duke University, United States of America

## Abstract

**Purpose:**

To develop a new three-dimensional (3D) spectral-domain optical coherence tomography (SD-OCT) data analysis method using a machine learning technique based on variable-size super pixel segmentation that efficiently utilizes full 3D dataset to improve the discrimination between early glaucomatous and healthy eyes.

**Methods:**

192 eyes of 96 subjects (44 healthy, 59 glaucoma suspect and 89 glaucomatous eyes) were scanned with SD-OCT. Each SD-OCT cube dataset was first converted into 2D feature map based on retinal nerve fiber layer (RNFL) segmentation and then divided into various number of super pixels. Unlike the conventional super pixel having a fixed number of points, this newly developed variable-size super pixel is defined as a cluster of homogeneous adjacent pixels with variable size, shape and number. Features of super pixel map were extracted and used as inputs to machine classifier (LogitBoost adaptive boosting) to automatically identify diseased eyes. For discriminating performance assessment, area under the curve (AUC) of the receiver operating characteristics of the machine classifier outputs were compared with the conventional circumpapillary RNFL (cpRNFL) thickness measurements.

**Results:**

The super pixel analysis showed statistically significantly higher AUC than the cpRNFL (0.855 vs. 0.707, respectively, p = 0.031, Jackknife test) when glaucoma suspects were discriminated from healthy, while no significant difference was found when confirmed glaucoma eyes were discriminated from healthy eyes.

**Conclusions:**

A novel 3D OCT analysis technique performed at least as well as the cpRNFL in glaucoma discrimination and even better at glaucoma suspect discrimination. This new method has the potential to improve early detection of glaucomatous damage.

## Introduction

Optical coherence tomography (OCT) is a rapidly evolving technology and has gained a significant clinical impact in ophthalmology.[1]–[4] One of the major OCT applications is glaucoma assessment by measuring retinal nerve fiber layer (RNFL) thickness in circumpapillary and macula regions. Many studies showed that the current OCT quantitative assessment has excellent glaucoma discriminating ability. [1], [3], [5], [6].

**Figure 1:**
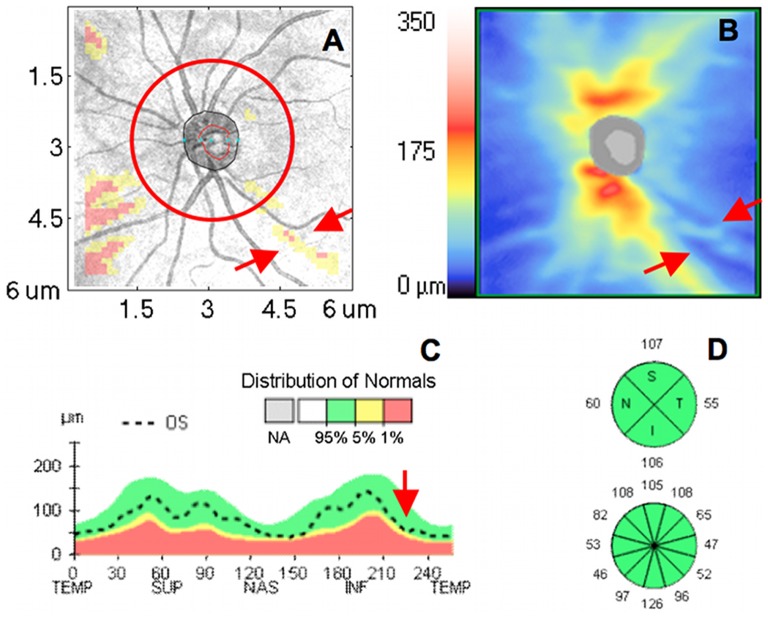
An example of conventional circumpapillary retinal nerve fiber layer (cpRNFL) analysis as provided by Cirrus HD-OCT. (A) Overlay of RNFL thickness deviation map on the OCT fundus image with focal wedge defect (red arrows) predominantly outside the 3.4 mm diameter circle sampling (red circle). (B) Corresponding 2D RNFL thickness map, RNFL focal defect is marked with red arrows. (C) cpRNFL thickness profile along the 3.4 mm diameter circle is within the normal range (green range). Red arrow pointing to the approximate location of the RNFL wedge defect. (D) The RNFL thickness measurement is summarized in 4 quadrants and 12 clock hours with all sectoral measurements within the normal range.

Spectral-domain (SD-) OCT’s fast scanning speed allows three-dimensional (3D) volume scanning of the retina, which may offer detailed and accurate quantitative analysis of the retinal structure. However, despite the rich information embedded in 3D OCT images, current standard quantitative structural OCT measurement is mostly limited to several hundred sampling points along a 3.4 mm circle diameter centered at optic nerve head, which does not take full advantage of the 3D dataset (over 20,000 sampling points). This sampling pattern was chosen mostly to allow compatibility with legacy data obtained using time-domain (TD-) OCT. The limited tissue sampling might lead to situations where early signs of structural changes are not detected when located outside the sampled circle (Fig. 1).

In addition to the circumpapillary RNFL (cpRNFL) thickness measurements, most SD-OCT devices provide the color coded thickness deviation map to highlight locations of structural damage (Fig. 1). These maps are generated by avaraging the mean RNFL thickness within a fixed number of neighboring sampling points (fixed-number super pixels) and comparing this measurement with age-matched normative databse. However, there is no quantitative summary of this super pixel analysis, and therefore clinicians’ subjective interpretation is required.

In the clinical practice, subjects are often classified into three major groups: healthy, glaucoma suspect or glaucoma. Classifying subjects as “suspect” has important clinical advantage because some of these subjects posses pre-perimetric glaucoma characteristics. With the paucity of functional indication of glaucoma, correctly identifying glaucoma suspect based on their structural features is of upmost importance as it would allow timely adjustemt of clinical management. However, detection of pre-perimetric disease is still posing a significant challenge. [7], [8] We hypothesize that a comprehensive use of the full 3D OCT data would improve detection of early glaucoma. The purpose of this study was to develop a novel 3D SD-OCT data analysis technique utilizing the full 3D dataset to improve the ability to detect glaucomatous structural damage at early stages. The new method uses variable-size super pixel mapping with a machine classifier analysis to quantitatively assess the full 3D OCT data. Unlike the conventional super-pixel analysis, which uses a fixed number of points, the newly developed variable-size super pixel is defined as a cluster of homogeneous adjacent pixels with variable size, shape and number. In this paper, we investigate whether the variable-size super pixel analysis is better suited for handling individual eye characteristics that might lead to improved glaucoma diagnosis predominantely in early disease stage.

## Methods

### Ethics Statement

This study was obtained the Institutional Review Board (IRB) approval named “Optical Coherence Domain Reflectometry and Optical Coherence Tomography Measurements of Intraocular Structure”.

### Subjects and Image Acquisition

This study was conducted with healthy, glaucoma suspect and glaucomatous eyes with a wide range of disease severity selected from the Pittsburgh Imaging Technology Trial (PITT). One hundred and ninety-two eyes of 96 subjects (44 healthy, 59 glaucoma suspect and 89 glaucomatous eyes) were enrolled to test the glaucoma discrimination performance. An independent dataset, including 46 eyes of 46 healthy subjects (randomly selected one eye for each subject), was used as the normative database. Institutional Review Board (IRB) approval was obtained for the study and all participants gave their consent to participate in the study. The study adhered to the Declaration of Helsinki and Health Insurance Portability and Accountability Act regulations.

Exclusion criteria for the study included history of ocular trauma or surgery (except for uncomplicated cataract or glaucoma surgery) and disease or treatment that might affect the visual field (e.g., stroke, central nervous sytem tumors) or retinal thickness (diabetes melitus, chronic steroid treatment).

All subjects had a comprehensive ophthalmic evaluation, reliable visual field (VF) and 3D SD-OCT scan all acquired within 6 months of each other. The ophthalmic evaluation included medical history, best-corrected visual acuity, manifest refraction, intraocular pressure (IOP) measurement, gonioscopy, slit-lamp examination before and after pupil dilation, and VF testing. VF was considered reliable if false negatives, false positives and fixation losses were less than 30%. All subjects had 3D OCT scans centered at the optic nerve head (ONH) (Cirrus HD-OCT; Carl Zeiss Meditec, Inc., Dublin, CA; ONH cube 200×200 scan protocol).

### Clinical Diagnosis

Eyes were defined as healthy if there was no history of glaucoma, IOP≤21 mmHg, the ONH did not meet the criteria for glaucomatous optic neuropathy (GON), as described below, and VF was normal.

Eyes were defined as glaucomatous if there was both GON and glaucomatous VF loss. GON was defined if either one of the following criteria was met: inter eye cup-to-disc (C/D) ratio asymmetry >0.2, accounting for disc size; rim thinning or notching; cup to disc ratio ≥0.6; RNFL wedge defect or disc hemorrhage. Glaucomatous VF was diagnosed if any of the following findings were evident on two consecutive VF tests: a glaucoma hemifield test outside normal limits, pattern standard deviation (PSD) <5%, or a cluster of three or more non-edge points in typical glaucomatous locations (arcuate scotoma, nasal step, paracentral scotoma or temporal wedge), all depressed on the pattern deviation plot at a level of p<0.05, with at least one point in the cluster depressed at a level of p<0.01.

Glaucoma suspect eyes had IOP between 22 to 30 mmHg and/or GON features all in the presence of a normal VF test.

### 3D SD-OCT Data Analysis

Super pixel mapping is a partitioning of image into a number of close-to-homogenous segments with variable sizes and shapes. The features of these variable-size super pixels were then extracted and used as the input for machine classifier analysis in order to generate a key index output for each image. The procedure to generate the index output included the following steps: comparison with normative database, feature map generation, super pixel mapping, feature extraction, and classification.

#### 1. Normative Database

**Figure 2:**
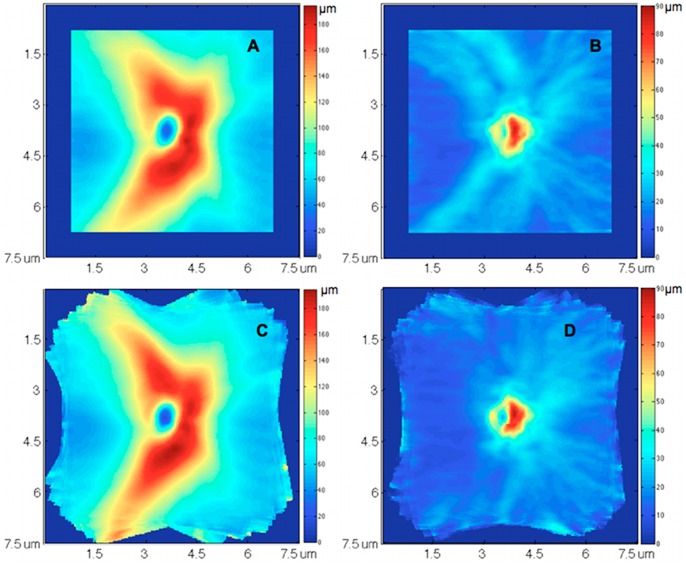
Normative database normalization with 46 healthy eyes. (A, B) mean and standard deviation (SD) of retinal nerve fiber layer (RNFL) thickness measurement at each sampling point (A-scan), without normalization. (C, D) mean and SD of RNFL thickness measurement after normalizing individual’s retinal nerve fiber bundle path location to population’s average location. The variations of RNFL thickness were larger at superior temporal and inferior temporal regions (brighter blue in B) because of the population variation of the bundle locations. After aligning the bundle locations and normalizing the RNFL thickness map, the RNFL thickness variations at these two regions were markedly reduced (dark blue in D).

A normative database was assembled to measure the RNFL thickness deviation at each sampling point in the 3D dataset. Retinal layer segmentation, which was optimized for 3D SD-OCT dataset [9] was applied on each 3D OCT image to obtain the RNFL thickness and reflectivity at every single sampling point (total 200×200 points). All segmented 3D OCT datasets were visually evaluated to ensure the correct segmentation. Any OCT scan with >8% consecutive B-scan from the total number of B-scans that showed segmentaion error or >12% cumulative segmentation error was excluded from the study. ONH margin was automatically detected on the OCT fundus image using a software program of our own design. [10] Major retinal nerve fiber bundle path at each hemi-field on each RNFL thickness map was automatically detected. Each RNFL thickness map was then normalized by aligning the bundle location at each hemi-field to a reference position (population’s average bundle location) for the comparison with the normative database in order to minimize spatial variability in RNFL thickness, especially at superior temporal and inferior temporal regions (Fig. 2). The RNFL thickness map normalization was processed on the concentrated circles with different radii started from the ONH center. For each subject, the ONH center was first aligned at a reference center point. At each concentrated circle, two average bundle location (superior and inferior) were computed from the normative database. Each subject’s RNFL thickness profile at the given circle was normalized by strenching/shrinking so the subject’s bundle location would coincide with the population average bundle location. The entire RNFL thickness map was normalized by repeating this process at all concentrated circles.

#### 2. 2D Feature Map Generation

**Figure 3:**
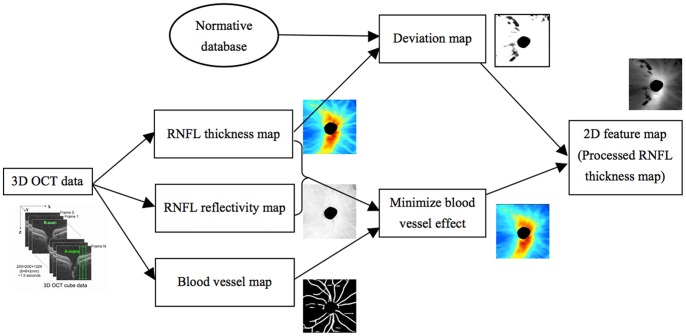
Flowchart of converting a 3D OCT image into a 2D feature map.

Each 3D OCT image (200×200×1024 voxels) was converted to a 2D feature map (200×200 pixels) as follows. RNFL thickness and reflectivity along with the blood vessel mapping were extracted from each image (Fig. 3). The normalized RNFL map after applying the bundle path correction were compared with the normative database point by point to obtain a deviation map, with the cut-off value set to mean value minus stardard deviation (SD). Unlike the conventional setting of cut-off value, i.e., mean value - 2SD in most OCT devices, this new setting is more sensitive to identify the case at the border of the normative database, which might be an indicator of the structural changes at the early stage. With this new setting, the RNFL data lower than the bottom 15.9% of the normative database was set with higher probability for the further process, comparing with the bottom 2.3% using the conventional setting. The internal reflectivity of retinal nerve fiber layer has been proved to be useful in glaucoma assessment. [11] The RNFL internal reflectivity, was calculated by taking the average reflectivity within the RNFL along each A-scan, with each voxel’s reflectivity normalized to its A-scan’s saturation before the averaging. Retinal blood vessel generated shadow at retina nerve fiber layer, which was noise in the RNFL thickness computation. Moreover, the vessel patterns varied randomly among subjects. To minimize the vessel effect on the RNFL thickness map, the retinal blood vessels in the 3D dataset were automatically detected using a 3D boosting algorithm [12] and filled out. The RNFL thickness of each pixel located at the blood vessel region was replaced by a value computed from all the non-vessel pixels on the RNFL thickness map using bi-linear interpolation. The final feature map is a function of the RNFL thickness and interal refleciviy after accounting for the blood vessls and the deviation map.

#### 3. Variable-size super pixel segmentation

Variable size/shape super pixels were automatically mapped on the 2D feature map by grouping homogeneous neighboring pixels using a ncut algorithm. [13] The ncut algorithm is to partition an image into dozens to thousands of small regions (called super pixels) by grouping neighboring pixels, where pixels within a partitioned region have homogenous properties while different partitioned regions have maximal differences in their properties. One hundred super pixels were initially segmented on the feature map. The size of each super pixel was automatically adjusted with the pre-defined criteria based on the pathologic contexts of glaucoma. To be more sensitive to RNFL thinning (glaucomatous damage), smaller super pixels were assigned to thinner RNFL. Each initially segmented super pixel was recursively partitioned into *N* more super pixels, while *N* was a function of mean, standard deviation, size, and deviation to the normative database of the given super pixel compared with global mean and standard deviation of the 2D feature map, written as:
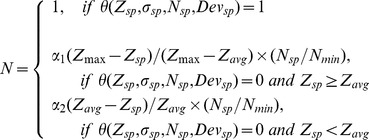
(1)where 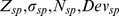 represented the average RNFL thickness, RNFL standard deviation, size and average deviation of the given super pixel, 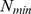 was a pre-defined minimal size of super pixel, 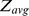 and 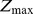 were the average and maximal thickness with the entire 2D RNFL thickness map. 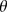 was defined with a series of criteria with “IF”, “logical AND”, “logical OR” and “logical NOT” operations, which corresponded to different super pixel conditions need further partition or not, written as:
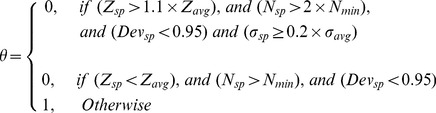
(2)

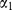
 and 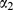
were two constants chosen based on the segmentation logic, which were used to control the number of segments partitioned in the given super pixel. For 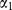, because super pixel RNFL thickness was thicker than the average RNFL thickness, this super pixel was further partitioned only if its standard deviation was large enough. We wanted the super pixel partitions into several big segments, and recursively partitions into small segments in the following iterations. Therefore, 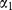 was set to 0.2 to lower the number of the segments. For 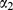, since the super pixel RNFL thickness was thinner than the average RNFL thickness, we wanted to partition the super pixel into many small segments. Therefore, 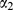 was set to 1.2 to higher the number of segments.

The stability is an important parameter in ncut algorithm to control the super pixel segmentation result. It was set to a small value, i.e., −0.1, to let the 100 initial super pixels have more flexible boundaries. In the recursive partition processing, to make the super pixels having smooth boundaries and compact shapes, the stability variable was set to a relatively large value, i.e. −0.006.

**Figure 4:**
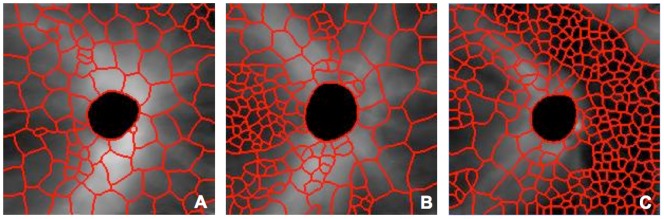
Super pixel segmentation on 3D OCT images. (A) Analysis output in a healthy eye, (B) glaucoma suspect, and (C) glaucomatous eye. Abnormally thin retinal nerve fiber layer is marked with small super pixels.

In summary, the super pixel number and size were automatically adjusted by initial segmentation and recursive partition. The segmented feature map provided a qualitative analysis with more natural representation, in which damaged areas tended to have smaller super pixels, while normal regions had larger super pixels (Fig. 4).

#### 4. Feature Extraction

Super pixel map provided a qualitative representation of the structural damage. To summarize the map as quantitative disease indices, a total of 68 super pixel features were extracted and used as the inputs of machine learning classifiers analysis (Table 1). Three thresholds, Tmin, T1, and T2 were set to 50, 133, and 400 pixels respecitvely based on the experiments to obtain two sub-groups of super pixel with large size and small size. The features were calculated from all super pixels together with two sub-groups.

**Table 1:** Super pixel features used as inputs for the machine learning classifier.

	Super Pixel Features	Healthy	Glaucoma Suspect	Glaucoma
**Number of super pixels**	Total number of super pixels	151.8±59.5	187.5±64.4	250.8±59.4
	Total number of small super pixels with “Tmin<size<T1”	66.1±54.0	97.0±55.1	150.9±54.3
	Total number of super pixels with “size>T2”	40.5±14.3	31.3±14.7	18.3±11.9
	Numbers of super pixels with the RNFLthickness at pre-defined ranges:			
	0–30 µm	41.4±53.0	67.1±56.4	139.3±75.8
	75–120 µm	26.3±7.4	26.6±7.0	16.5±9.6
	120–165 µm	4.5±3.1	1.4±2.0	0.6±1.2
	Numbers of super pixels with the size atpre-defined ranges:			
	30–120 pixels	65.9±54.0	98.1±56.0	152.7±53.5
	360–420 pixels	7.1±4.4	5.0±3.3	3.7±2.5
	420–540 pixels	15.4±7.6	11.5±6.5	6.5±4.4
**Mean, Standard** **deviation, 3^rd^ and 4^th^** **central moments**	Super pixel RNFL thickness			
		55.9±15.1 µm	48.2±12.9 µm	34.8±10.9 µm
		27.4±4.0 µm	24.99±2.8 µm	21.8±4.0 µm
		0.9±0.3	0.89±0.2	1.19±0.4
		3.2±0.8	3.19±0.7	4.29±1.8
	Super pixel size			
		288.8±102.9	231.1±91.5	163.3±50.5
		204.2±42.9	191.3±53.2	141.9±54.3
		0.9±1.2	1.7±1.1	2.5±0.8
		4.8±4.3	7.1±5.2	11.8±6.3
	Super pixel RNFL thickness with“Tmin<size<T1”			
		47.1±22.8 µm	40.0±16.0 µm	26.6±9.2 µm
		21.7±7.5 µm	21.2±6.6 µm	17.6±3.9 µm
		1.3±0.8	1.4±0.6	1.8±0.5
		5.4±3.4	5.5±2.3	7.7±3.0
	Super pixel RNFL thickness with “size >T2”			
		70.9±11.7 µm	65.2±8.6 µm	52.3±13.1 µm
		27.0±5.5 µm	22.8±3.5 µm	18.1±7.0 µm
		0.6±0.2	0.6±0.4	0.8±0.5
		2.4±0.4	2.6±0.8	2.9±1.3
**Distributions**	The value of each bin in the normalizedhistogram distribution of super pixelRNFL thickness	Figure 5A	Figure 5A	Figure 5A
	The value of each bin in the normalizedhistogram distribution of super pixel size	Figure 5B	Figure 5B	Figure 5B
**Average RNFL thickness**	Thickness at 3.4mm circle	105.3±18.6 µm	96.1±15.5 µm	72.3±19.3 µm
	Thickness at entire scan region	89.5±15.9 µm	79.0±13.3 µm	61.8±15.1 µm
	Thickness at entire scan region excludingONH region	88.0±15.6 µm	78.4±13.5 µm	61.1±15.2 µm

Data reported as Mean ± SD.

Tmin,T1, T2– Thresholds of minimal super pixel size, small super pixel size and large super pixel size respectively, RNFL – retinal nerve fiber layer, ONH – optic nerve head.

#### 5. Glaucoma Classification

Glaucoma classification was performed by implementing LogitBoost adaptive boosting algorithm, [14] which was designed as a supervised two-class machine classifier. Because the machine classifier was a two-class classifier, only two of the three clinical groups (healthy, glaucoma suspects, and glaucoma) were used to train the classifier each time. The output of the classifier was a continuous number ranging from negative (health) to positive (disease) value. Ten-fold cross validation was used to train and test the machine classifier.

### Statistical Analysis and Evaluation

The software performance was evaluated by the area under the receiver operating characteristics (AUCs) of the machine classifier outputs, for discriminating between healthy and diseased eyes. AUCs were compared with those of the conventional method of diagnosis – the mean and best quadrant measurements of the circumpapillary RNFL (cpRNFL) thickness generated by Cirrus HD-OCT software. If both eyes had the same clinical diagnosis, one eye was randomly selected from each subject to compute AUCs. AUCs were compared using the Jackknife method, [15] which is equivalent to the method of DeLong et al. [16] Sensitivity at the 85% specificity was calculated for each of the machine classifier outputs and compared with the measurements of conventional cpRNFL analysis.

## Results

One hundred and ninety-two eyes of 96 subjects (44 healthy, 59 glaucoma suspect and 89 glaucomatous eyes) qualified for the study. The characteristics of the study participants were summarized in Table 2. Five glaucomatous eyes (5 subjects; 5.2%) were excluded from the study due to retinal layer segmentation failure.

**Table 2:** Characteristics of the study participants.

	Healthy*n* = 44	GlaucomaSuspect*n* = 59	Glaucoma*n* = 84	p-value
**Age (years)**	54.8±8.9	61.9±9.6	66.9±8.7	<0.001*
**Male/female**	5/18	13/24	21/30	0.269^†^
**MD (dB)**	−0.09±1.28	−0.45±1.64	−5.88±5.80	<0.001^‡^

Values are means ± standard deviation.

MD – visual field mean deviation, * –ANOVA with linear model, ^†^ – Chi square test, ^‡^ – ANOVA with mixed effects models.

**Figure 5:**
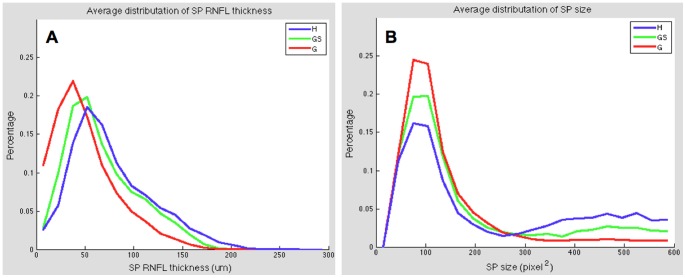
Normalized histogram distribution of super pixel. (A) RNFL thickness and (B) super pixel size for healthy (H), glaucoma suspect (GS), and glaucomatous (G) eyes.

Examples of the results of super pixel mapping are given in Fig. 4. The super pixel boundaries were superimposed on the processed grey scale RNFL thickness map, where a brighter pixel intensity corresponded to a thicker RNFL. Different super pixel distribution patterns are evident in the various stages of glaucomatous damage. The population’s average distributions of super pixel RNFL thickness and size for healthy, glaucoma suspects, and glaucomatous eyes are illustrated in Fig. 5.

**Figure 6:**
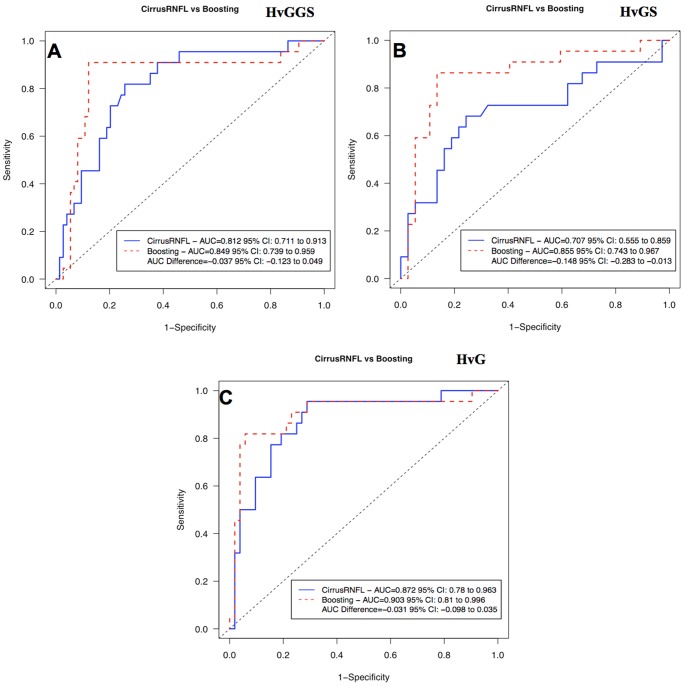
The receiver operating characteristic curves (ROCs) computed with the machine classifier method and Cirrus HD-OCT software generated mean cpRNFL thickness. (H) healthy eyes, (G) glaucomatous eyes, (GS) glaucoma suspect eyes.

Glaucoma discriminating performance was assessed with three different grouping combinations: healthy vs glaucoma+glaucoma suspects (HvGGS), healthy vs glaucoma suspects (HvGS), and healthy vs glaucoma (HvG). Table 3 gives the performance of cpRNFL thickness measurements in global and 4 quadrants. Comparing with the conventional cpRNFL thickness, machine classifier provided better AUCs and higher sensitivities (Table 4 and Fig. 6). The AUC for HvGS was statistically significantly higher with the super pixels analysis than the conventonal cpRNFL thickness (0.855 vs 0.707, respectively, p = 0.031, Jackknife test), while no significant difference was detected with HvGGS and HvG. Comparing with the best quadrant measurements, i.e., the inferior quadrant, the machine classifier showed higher sensitivities for HvGGS and HvGS without reaching statistical significance (Table 5).

**Table 3:** Area under the receiver operating characteristic curves (AUCs) of conventional circumpapillary RNFL (cpRNFL) thickness measurements in global and four quadrants from Cirrus HD-OCT software.

	Retinal nerve fiber layer thickness				
	Mean	Temporal	Superior	Nasal	Inferior
**HvGGS**	0.812	0.638	0.781	0.604	0.862
**HvGS**	0.707	0.598	0.639	0.519	0.807
**HvG**	0.872	0.656	0.844	0.614	0.916

HvGGS – healthy vs glaucoma+glaucoma suspects, HvGS – healthy vs glaucoma suspects, HvG – healthy vs glaucoma.

**Table 4:** Area under the receiver operating characteristic curves (AUCs) computed with machine classifier and Cirrus HD-OCT software generated mean cpRNFL thickness.

	cpRNFL thickness		Boosting machine classifier		
	AUC	Sensitivity at the 85% Specificity	AUC	AUC Difference† [95% CI]	Sensitivity at the 85% Specificity
**HvGGS**	0.812	45.5%	0.847	0.035 [−0.053, 0.122]	90.9%
**HvGS**	0.707	45.5%	0.855	0.148* [0.013, 0.283]	86.4%
**HvG**	0.872	63.6%	0.903	0.031 [−0.035, 0.098]	81.8%

† Difference with RNFL thickness AUC, * statistically significant.

HvGGS – healthy vs glaucoma+glaucoma suspects, HvGS – healthy vs glaucoma suspects, HvG – healthy vs glaucoma, CI – confidence interval.

**Table 5:** Area under the receiver operating characteristic curves (AUCs) computed with machine classifier and Cirrus HD-OCT software generated cpRNFL thickness in inferior quadrant.

	Inferior cpRNFL thickness		Boosting machine classifier		
	AUC	Sensitivity at the 85% Specificity	AUC	AUC Difference† [95% CI]	Sensitivity at the 85% Specificity
**HvGGS**	0.862	72.7%	0.847	−0.015 [−0.097, 0.066]	90.9%
**HvGS**	0.807	72.7%	0.855	0.048 [0.093, 0.189]	86.4%
**HvG**	0.916	81.8%	0.903	−0.013 [−0.082, 0.056]	81.8%

† Difference with inferior RNFL thickness AUC.

HvGGS – healthy vs glaucoma+glaucoma suspects, HvGS – healthy vs glaucoma suspects, HvG – healthy vs glaucoma, CI – confidence interval.

## Discussion

Current standard quantitative glaucoma analysis using 3D SD-OCT have substantial room for improvement especially for detection of early glaucoma. [7], [8] One possible reason is that only a small fraction of the 3D dataset is used in the analysis. We suggest a new 3D OCT data analysis method, using machine learning technique based on variable-size super pixel mapping to quantitatively summarize the full 3D dataset and automatically identify glaucomatous eyes. We demonstrated that this method was better at discriminating glaucoma suspect eyes from healthy eyes (HvGS) comparing with mean cpRNFL thickness, and performed at least as well for HvG and HvGGS. The improved performance in discriminating early disease is because this method is tailored to detect localized damage that typically occurs at early stage of glaucoma. However, in late stage of the disease, a globalize damage is more common where the variably sized super pixel analysis has the same performance as the cpRNFL analysis.

There are several advantages of variable size/shape super pixel machine classifier analysis. First, flexible size and shape super pixel processing provides more natural representation to fit the variable spatial architecture of the structural damages. Many ocular diseases demonstrate areas of pathologic change with measureable differences from unaffected areas when considering features such as retinal layer thickness, internal reflectivity, etc.[6], [17]–[19] These pathologically affected areas usually share similar characteristics but with variable magnitude in variable shape and size. Therefore, variable-size super pixel segmentation efficiently depict this natural representation by grouping similar neighboring sampled points based on the homogeneity of various features. Second, super pixel processing is computationally efficient. Super pixel mapping reduces the number of data points from over 20,000 sampling points down to a few hundred super pixels by condensing the redundant information. This is a common approach for handling such a high density data with the balance of preserving meaningful information and improving the computational efficiency. Moreover, machine classifier provides an efficient and optimized way to combine dozens of super pixel features together with global RNFL features into one key index to automatically identify diseased eyes. The performance of the software was based on the combination of super pixel processing and machine learning technique. Our study showed that the new super pixel analysis method quantitatively summarize the full 3D OCT dataset, which outperformed the conventional cpRNFL analysis in terms of discriminating ability and diagnostic sensitivity for early glaucoma detection.

To make this super pixel analysis sensitive to the localized damages, we set a criterion of super pixel mapping so that smaller super pixel corresponded to the thinner RNFL. With the natural distribution of the retinal nerve fiber, the RNFL is thicker in superior-temporal and inferior-temporal areas and thinner in temporal and nasal areas. In our method, the deviation from the normative database was used to partially control the super pixel mapping (Equation 1 and 2). Therefore, although temporal and nasal areas had relatively thinner RNFL thickness, they would not have smaller super pixels if the RNFL thickness was within the range of the normative database.

In the glaucoma classification step, both local features (super pixel feautres) and glocal features (global RNFL measurements) were used to train and test the boosting machine classifier. Global featuares, such as cpRNFL, were able to discriminate most glaucomatous eyes. Local features were able to enhance localized glaucomatous damages for eyes at the early stages of the disease. Therefore, combining both glocal and local featuares in the machine classifier improved the diagnostic sensitivity comparing with the conventional cpRNFL measurement. Including more features and applying feature selection operation may further enhance the performance of the machine classifier.

Searching the literature, we were able to find only one publication where glaucoma strctural analysis used the information from the entire 3D dataset. [7] In that study, 2D RNFL deviation map was analyzed and subjectively graded into 5 different groups. Compared with conventional cpRNFL analysis, this analysis provided additional spatial and morphologic information of RNFL damage, and significantly improved the diagnostic sensitivity for glaucoma detection. This is consistent with the result of our super pixel analysis study that analyzed the full 3D dataset providing more localized and detailed information of structural damages, and showing the potential to detect structural damage in early stages of the disease. The advantages of our method are the fully automated process and advanced data analysis compared to the subjective grading system used in the previous study. The expansion of this analysis will be including the distribution, shape and spatial locations of the localized structural changes with the super pixel and machine classifier analysis, which may further improve the performance of the 3D data analysis.

In this study we used only one SD-OCT device out of a variety of devices that are commercially available. However, the conceptual approach can be applied to any of these devices.

In conclusion, the super pixel processing with machine classifier analysis generated from 3D SD-OCT data has the potential to improve early detection of glaucomatous structural damages. This method can be easily extended to other ocular diseases by modifying the features corresponding to the various pathologic contexts.
